# Statistical analysis plan for the stepped wedge clinical trial Healing Right Way—enhancing rehabilitation services for Aboriginal Australians after brain injury

**DOI:** 10.1186/s13063-022-06800-0

**Published:** 2022-10-22

**Authors:** Elizabeth Armstrong, Tapan Rai, Deborah Hersh, Sandra Thompson, Juli Coffin, Natalie Ciccone, Leon Flicker, Dominique Cadilhac, Erin Godecke, Deborah Woods, Colleen Hayward, Graeme J. Hankey, Meaghan McAllister, Judith Katzenellenbogen

**Affiliations:** 1 School of Medical and Health Sciences, Edith Cowan University, 270 Joondalup Drive, Joondalup, WA 6027 Australia; 2 School of Mathematical and Physical Sciences, University of Technology Sydney, Broadway, Australia; 3 Curtin School of Allied Health, Curtin University, Perth, Australia; 4 Western Australian Centre for Rural Health, University of Western Australia, Geraldton, Australia; 5 Telethon Kids Institute, Broome, Australia; 6 Ngangk Yira Research Centre for Aboriginal Health and Social Equity, Murdoch University, Broome, Australia; 7 Medical School, University of Western Australia, Perth, Australia; 8 School of Clinical Sciences, Monash University, Melbourne, Australia; 9 School of Medical and Health Sciences, Edith Cowan University and Sir Charles Gairdner and Osborne Park Healthcare Group, Perth, Australia; 10 Geraldton Regional Aboriginal Medical Service, Geraldton, Australia; 11 Centre for Indigenous Australian Education and Research, Kurongkurl Katitjin, Australia Edith Cowan University, Perth, Australia; 12 Medical School, Faculty of Medical and Health Sciences, University of Western Australia, Perth, Australia; 13 School of Medical and Health Sciences, Edith Cowan University, Perth, Australia; 14 School of Population and Global Health, University of Western Australia, Perth, Australia

**Keywords:** Brain injury, Stroke, Aboriginal, Rehabilitation, Statistical analysis plan, Stepped wedge randomised control trial

## Abstract

**Background:**

Aboriginal Australians are known to suffer high levels of acquired brain injury (stroke and traumatic brain injury) yet experience significant barriers in accessing rehabilitation services. The aim of the Healing Right Way trial is to evaluate a culturally secure intervention for Aboriginal people with newly acquired brain injury to improve their rehabilitation experience and quality of life. Following publication of the trial protocol, this paper outlines the statistical analysis plan prior to locking the database.

**Methods:**

The trial involves a stepped wedge design with four steps over 3 years. Participants were 108 adult Aboriginal Australians admitted to one of eight hospitals (four rural, four urban) in Western Australia within 6 weeks of onset of a new stroke or traumatic brain injury who consented to follow-up for 26 weeks. All hospital sites started in a control phase, with the intervention assigned to pairs of sites (one metropolitan, one rural) every 26 weeks until all sites received the intervention. The two-component intervention involves training in culturally safe care for hospital sites and enhanced support provided to participants by Aboriginal Brain Injury Coordinators during their hospital stay and after discharge. The primary outcome is quality of life as measured by the Euro QOL–5D-3L VAS. A mixed effects linear regression model will be used to assess the between-group difference at 26 weeks post-injury. The model will control for injury type and severity, age at recruitment and time since commencement of the trial, as fixed effects. Recruitment site and participant will be included as random effects. Secondary outcomes include measurements of function, independence, anxiety and depression, carer strain, allied health occasions of service received and hospital compliance with minimum processes of care based on clinical guidelines and best practice models of care.

**Discussion:**

The trial will provide the first data surrounding the effectiveness of an intervention package for Aboriginal people with brain injury and inform future planning of rehabilitation services for this population. The statistical analysis plan outlines the analyses to be undertaken.

**Trial registration:**

Australia New Zealand Clinical Trials Registry ACTRN12618000139279. Registered 30 January, 2018.

## Background

Brain injury resulting from both stroke and traumatic injury can have significant ongoing effects on survivors in terms of affecting their sense of self, social relationships, mental health and community participation including employment prospects [1, 2]. Family members’/carers’ quality of life can also be affected as their lives change when supporting the person with the brain injury [3]. For Aboriginal Australians, as for other Indigenous populations, stroke occurs at a higher rate than for their non-Indigenous counterparts, and at a relatively younger age, reflecting the high prevalence of comorbidities such as diabetes and chronic cardiovascular conditions [4–9]. Traumatic brain injury (TBI) is also known to occur at a higher rate [10, 11]. Previous research has highlighted mismatches between community needs and services currently offered to Aboriginal people with brain injury and their families/community [12–14]. This has resulted in limited ongoing engagement between Aboriginal people with brain injury and hospital-based rehabilitation services, with particular challenges for (although not limited to) those living in rural and remote areas. Aboriginal people with brain injury have highlighted the need for (i) services that respect and incorporate cultural protocols and values, (ii) culturally secure assessment and treatment tools, (iii) support after hospital discharge and (iv) Aboriginal health worker involvement in this support.

Healing Right Way is a stepped wedge trial that implements and assesses the impact of a research-informed culturally secure [15] intervention model for Aboriginal people with brain injury in eight hospital sites in Western Australia (WA). The trial is registered in the Australia New Zealand Clinical Trials Registry (ACTRN12618000139279) and has received multiple relevant ethics approvals. The main trial protocol [16] was published previously. The current version of the trial protocol is Version 5 dated 3/11/20. This document updates and extends the statistical analyses outlined in the protocol and describes the presentation of results for the principal paper(s) which will report the results of the primary and secondary effectiveness hypotheses.

## Study overview

### Aims and hypotheses

The primary hypothesis is:

- H1. Compared to usual care (UC), implementation of the proposed intervention package (IP) will result in a higher score on the Euro QOL–5D-3L [17] VAS at 26 weeks post-injury.

The secondary hypotheses are:

- H2. Compared to UC, implementation of the IP will result in an improvement in service delivery at 12 and 26 weeks post-injury as evidenced by increased occasions of service.
- H3. Compared to UC, implementation of the IP will result in an improvement in service delivery at 12 and 26 weeks post-injury as related to increased compliance with minimum process of care indicators.
- H4. Compared to UC, implementation of the IP will result in significant reduction in disability (Modified Rankin Scale, mRS) [18] and greater independence (Functional Independence Measure, FIM™) [19] at 12 and 26 weeks post-injury.
- H5. Compared to UC, implementation of the IP will result in significantly less carer burden (Modified Caregiver Strain Index) [20] and less brain injury survivor anxiety and depression (Hospital Anxiety and Depression Scale) [21] at 12 and 26 weeks post-injury.
- H6. The culturally sensitive IP will be more cost-effective (additional benefits gained will justify additional costs for delivering the intervention; may lead to potential cost-offsets from less severe disease) than UC 26 weeks post-injury.
- H7. The IP will be acceptable to health professionals and Aboriginal participants and their families, and the role of the Aboriginal Brain Injury Coordinator is a feasible one.

The primary hypothesis (H1) and the first four of these (H2–H5) will be the focus of this statistical analysis plan. Cost effectiveness (H6) will be assessed by the health economics expertise within the trial team and will be published separately. Acceptability and feasibility of the Aboriginal Brain Injury Coordinator intervention (H7) will be assessed using qualitative analysis methods as per the published protocol for the project’s Process Evaluation [22].

### Study design

**Fig. 1:**
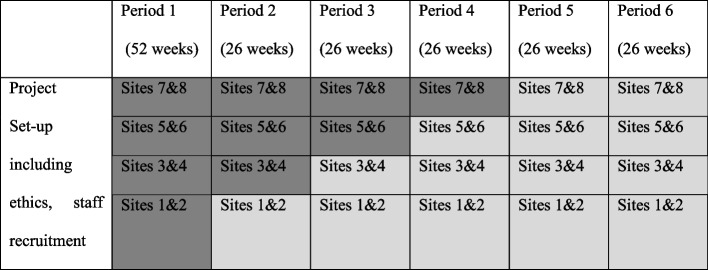
Implemented stepped wedge design

Healing Right Way was planned as a stepped wedge trial with four steps over 3 years. Eight sites (four rural hospitals and four urban sites) are participating in the trial. The 3-year timeframe was divided into six 26-week periods. At the start of the trial, all sites were in the control condition which contributed a baseline measurement for all sites. The trial plan called for two sites cross over to the intervention condition, at the end of the first 26-week period, while the other sites remained in the control condition. At the end of the second 26-week period, two additional sites were to cross over from the control condition to the intervention condition, and at the end of the third 26-week period, the last two sites were to cross over to the intervention condition. Starting with the fifth 26-week period, all sites were to be in the intervention condition. The trial design allowed for one additional 26-week period in which all sites were to be in the intervention condition. We recruited fewer people than anticipated—especially in the initial periods after the trial commenced. The trial design was therefore modified to accommodate the reduced recruitment numbers. On advice of the Trial Management Committee, the trial design maintained four steps as planned, but the baseline extended period by an additional 26 weeks. The modified design is presented in Fig. 1.

Participants were initially recruited within 4 weeks post-injury. This was amended during Period 1 to 6 weeks post-injury to enable recruitment of otherwise eligible patients who were only referred to the study immediately prior to the 4-week cut-off and for whom the recruitment process could not be organised within the short time frame. Outcome data were collected from participants and caregivers at 12 weeks and 26 weeks post-injury (as outlined in Table 1 below). Baseline assessments were predominantly conducted at the trial site at the time of recruitment. However, an amendment during Period 1 enabled recruitment in the community, to enable participants who were only referred to the study immediately prior to hospital discharge to be recruited. Participants were generally expected to have been discharged from the hospital sites prior to the 12-week and 26-week assessments. These assessments were therefore predominantly conducted off-site.

**Table 1:** Schedule for collection of participant-level outcome measures

Data collection point (*time post-injury)*	Baseline (*0–6 weeks)*	1 (*12 weeks)*	2 (*26 weeks)*
Demographic data	X		
Type of injury (TBI/stroke)	X		
EuroQoL-5D-3L		X	X
mRS	X	X	X
FIM™	X	X	X
HADS	X	X	X
Participant satisfaction		X	X
Modified Caregiver Strain Index (administered to participant’s caregiver)		X	X

### Randomisation of sites

Since the rural sites are smaller in size and were expected to recruit fewer participants, a restricted randomisation strategy was considered appropriate. Moreover, due to differences in the availability of advanced care in the four rural sites, patients are often transferred from these sites to one of the urban sites. Therefore, for the purpose of randomisation, each rural site was paired with the urban site to which it usually transfers patients for acute care as needed. The four pairs formed by this process were treated as clusters for the purposes of randomisation. These clusters were designated as A, B, C or D by the trial data and operations manager. The trial statistician who was blinded to this designation, then generated the sequence in which the clusters would cross over to the intervention condition, using simple randomisation.

### Recruitment and allocation of participants

The trial used a continuous recruitment strategy [23, 24]. All eligible participants who presented to a participating site in the trial period were invited to participate. Participants who accepted the invitation were allocated to the trial intervention phase (control or intervention) that the site was undergoing at the time of their recruitment.

### Patient population

The patient population consisted of Aboriginal Australians, 18 years or older who presented at one of eight hospital sites in Western Australia during the trial period after having experienced either an acute stroke or traumatic brain injury and were within 6 weeks post event. Participants had to have a neurological deficit present as reflected on the NIHSS [25] (stroke patients). Patients with TBI could not have a Glasgow Coma Scale (GCS) [26] of < 8. All had to be deemed to be able to benefit from rehabilitation by their medical and allied health team. Further detailed inclusion and exclusion criteria are provided in the published study protocol [16].

### The intervention

The trial implemented a complex intervention consisting of cultural security training for hospital staff and the employment of Aboriginal Brain Injury Coordinators (ABIC) to see the participants in hospital and up until 26 weeks post-injury onset and provide education, support, liaison and advocacy services to the participants and their families. The cultural security training involved workshops training 20 health professionals at each site (nursing, medical and allied health staff). The workshops were offered every 6 months during the intervention period for the site and consisted of a 3-h face to face component and 3 h of online modules. The Aboriginal Brain Injury Coordinator component required the Coordinator to be in contact with the participant a minimum of six times following their injury on a monthly basis during the intervention period.

### Sample size

Our sample size estimation procedure involved three steps. In the first step, we conducted a naïve sample size estimation which ignored the clustering effects of data collected through the stepped wedge design. In the second step, we calculated the design effect of the stepped wedge, using the method presented by Woertman et al. [27]. We then calculated the total sample size required by multiplying the naïve sample size calculated in the first step by the design effect and the total number of time periods in the planned design.

Our estimation was based on our primary outcome measure of Euro QOL–5D [17] VAS. We did not have any studies or preliminary data on the use of this measure in Aboriginal Australian populations. Therefore, our estimate of the effect of the intervention was based on the mean difference on the Euro QOL–5D [17] VAS between stroke and non-stroke populations in the published literature. Based on the literature [28, 29], the mean difference between stroke and non-stroke populations on the Euro QOL–5D [17] VAS was approximately 25 points with a standard deviation of 25. Guided by this information, we assumed that the intervention would result in an improvement of 15 points on the Euro QOL–5D [17] VAS (with a standard deviation 25), which equates to a standardised effect size of d = 0.6 for an independent sample t-test.

Using GPower 3.1 [30], we estimated the naïve sample size for an independent samples t-test and calculated that we would require a total of 90 participants to detect our estimated difference of d = 0.6 with 80% power at the 5% significance level.

The design effect proposed by Woertman et al. [27] is based on the number of clusters, number of steps, number of measurements at the end of each step and number of baseline measurements. Our proposed design had four clusters which were randomised to commence intervention in four steps, one baseline measurement and one measurement at the end of each step. We assumed a conservative intraclass correlation of 0.08. Based on these inputs, we calculated a design effect of 0.56. Multiplying the naïve sample size (n = 90) by the design effect (DEsw = 0.56) and the number of time periods in the trial (n = 24), and rounding-up our results to ensure that the number of patients per cluster per time period was an integer, we obtained a total sample size of 312 or 13 per cluster per time period.

We note that this approach may have over-estimated the required sample size since our design:

1. Incorporates an extra follow-up period which is not accounted for in the estimation.
2. Calls for two measurements for each individual (at each step): one at 12 weeks and the second at 26 weeks
3. Includes a round-up error by ensuring an integer value of participants in each cluster in each time period.

However, since we had little or no prior information in our estimates of effect size and intraclass correlation coefficient, this conservative approach in our estimation of total sample size was appropriate. Moreover, our estimate allowed for a 4% drop-out rate. Due to pragmatic considerations including the sparse distribution of the trial population in remote geographical regions, the estimated sample size was believed to be the maximum that could be achieved within the trial timeframe. Therefore, no additional allowance for drop-out was included in our sample size estimation.

### Blinding

All assessors were independent of the researchers involved in the intervention or the trial. Since the implementation of the intervention involved providing the treating clinicians with cultural security training, blinding of the treating clinicians was not possible. Similarly, since the intervention required the participant and/or the participant’s next of kin to interact with the ABIC, it was expected that the participants would be aware if they were receiving intervention. Therefore, the participants are not considered to have been blinded.

### Assessments

Participants were assessed at three timepoints on the outcome measures outlined below—within 6 weeks of injury and at 12 and 26 weeks post-injury (see Table 1).

Demographic data, injury type and severity, functional independence measures and hospital anxiety and depression were recorded at the baseline assessment.

### Primary outcome measure

The primary endpoint is the Euro QOL–5D-3L [17] VAS at 26 weeks post-injury.

### Secondary participant-level outcome measures

#### Participant outcome measures

The secondary participant-level outcome measures are:

- The modified Rankin Scale (mRS) [18]
- The Functional Independence Measure (FIM) [19]
- The Hospital Anxiety and Depression Scale(HADS) [21]
- Participant satisfaction

In addition, the Euro QOL–5D-3L [17] VAS at 12 weeks post-injury is considered a secondary outcome which will be reported in descriptive statistics but is not the subject of any hypotheses.

#### Caregiver outcome measures

The Modified Caregiver Strain Index [20] was administered at 12 weeks and 26 weeks post-assessment.

#### Other participant-level data

Demographic data collected at time of recruitment includes:

- Age
- Gender
- Recruitment site
- Living arrangements prior to injury
- Stroke/brain injury risk factors, e.g. hypertension, diabetes, alcohol consumption, adverse events (AEs) and serious adverse events (SAEs) that occurred while a participant was enrolled in the trial were recorded when notified.

#### System-level outcome measures

The health system-related outcomes recorded in this trial are:

- Minimum Processes of Care administered for each participant (including, e.g. use of interpreters, involvement of Aboriginal Liaison Officer, discharge plan involving family)
- Occasions of service
- Resource utilisation using a purposefully designed questionnaire completed by each participant

These data are collected according to the schedule in Table 2 below:

**Table 2:** Schedule for recording of system-level outcome measures

Data collection point (*time post-injury)*	Baseline (*0–6 weeks)*	1 (*12 weeks)*	2 (*26 weeks)*
Process of care indicators		X	X
Occasions of service		X	X
Resource utilisation		X	X

Resource utilisation will be used for the economic evaluation [16]

### Data management

Data collected during the trial will be recorded and stored using the RedCap™ [31] electronic data capturing tool. Additional data on outpatient use of allied health services during the trial period will be obtained from the Western Australia Department of Health (WA DoH). Data on inpatient use of allied health services during the trial will be obtained from the Allied Health System (AHS) where possible and manual extraction from medical files when not. In addition, data on Minimum Processes of Care will be extracted manually from files at the participating hospital sites. These data will be stored in comma separated values (csv) files, which will be merged with the trial data from RedCap™ [31] during analysis. All data and scripts generated for analysis will be archived and stored at the lead institution (Edith Cowan University) for a period of 7 years. Access to this data will be controlled by the lead chief investigator.

## Statistical analysis plan

### Analysis principles and general considerations

- All outcomes and analyses were prospectively characterised as primary or secondary.
- This is a superiority trial with intervention expected to yield superior outcomes, compared to control. However, all outcomes will be tested independently at the two-tailed 5% significance level. All estimates of treatment effects will be presented with 95% confidence intervals
- No formal adjustments will be undertaken to constrain the Type I error associated with planned secondary or exploratory analyses. The information provided by analyses is designed to supplement the evidence from the primary analyses; it will provide a more complete characterisation of the treatment effects.
- The analyses for all quantitative outcome measures will be conducted on an intention the treat (ITT) basis, i.e. all participants will be analysed in the trial phase (control or intervention) in which they were recruited, regardless of whether their treatment adhered to the trial protocol or not. The ITT strategy for this trial is based on the following principles:
  - ◦ All available outcome data are collected on all recruited participant
  - ◦ For the ITT analysis, the outcomes for each participant will be included in the data for the trial phase (control/intervention) in which the participant was recruited.
  - ◦ All available outcome data will be used in the primary analyses. The primary analyses will be reported without imputation of missing data. If the amount of missing data exceeds 10% at the primary endpoint, missing data will be imputed under the assumption that data is missing at random.
  - ◦ A sensitivity analysis including all randomised individuals will be conducted. The sensitivity analysis will consider alternative assumptions about data missing not at random (MNAR).
- For primary and secondary analyses, the treatment effects for the participant-level effectiveness outcomes will be adjusted for brain injury type (stroke or TBI) and severity of injury (mRS) [18] at baseline. In addition, age at recruitment, timepoint (time since injury) and time period (6-month block of time in which the participant is recruited to the step-wedge trial) will be included as fixed effects. Hospital site will be included as a random effect. Unadjusted analyses will be reported separately from these pre-specified analyses.
- All analyses will be conducted using the R Statistical Programming Language [32] after data collection is completed and the database is locked.

### Interim analysis

No interim analyses were planned for this trial. The Data and Safety Monitoring Committee (DSMC) reviewed safety and effectiveness data during the course of the trial. Due to the sequential nature of the trial (control condition precedes intervention condition at each site), there were no formal stopping conditions for effectiveness. The DSMC was guided by the Haybittle-Peto boundaries [33] in assessing safety concerns. That is, they could have recommended stopping of the trial if the number of serious adverse events (SAEs) in the intervention condition exceeded the number of SAEs in the control condition by at least 3 standard errors.

### Trial profile

The trial will be reported in accordance with the CONSORT extension for stepped wedge cluster randomised trials [23]. A CONSORT Flow Diagram will depict the total number of people screened for the trial and reasons for exclusion. Numbers related to withdrawal/loss to follow-up will be included in the diagram, with reasons detailed in text form.

### Demographics and baseline characteristics

Participant demographic and baseline clinical characteristics will be reported for participants recruited in each condition (control or intervention). Demographic characteristics will include age at recruitment, gender, recruitment region (metropolitan vs regional), place of residence (urban area, inner regional, outer regional, remote, very remote as per the Accessibility/Remoteness Index of Australia ARIA +) [34] and living arrangements prior to hospital admission. Baseline clinical characteristics will include injury type, injury severity (GCS [26] for TBI, NIHSS [25] for stroke and mRS [18] for all), HADS [21], FIM [19], history of alcohol consumption and drug use. Comorbidities/risk factors such as diabetes, renal and heart disease will be included. Categorical variables will be summarised using frequencies and percentages. Unless otherwise indicated in the tables, percentages will be based on the number of patients for whom data are available. Continuous variables will be summarised by the mean and standard deviation (SD) or by the median and interquartile range (IQR). The format for presentation of this data is outlined in Table 3 of Appendix.

### Adherence to intervention protocol

The number and percentage of participants who received the services of the ABIC will be reported. In addition, the number and percentage of participants who received the minimum number of ABIC visits (six visits on a monthly basis) as recommended in the protocol will be reported. In terms of the ABIC service, the per protocol groups will be formed based on whether participants adhered to the stated protocol or not—those who received the minimum amount of intervention will be included in the intervention group and those who did not receive the stated minimum amount of intervention, will be included in the control group. All hypotheses will be re-tested using the same models and standards as stated for the ITT analysis. The cultural security training will be reported in terms of a simple statement of the number of sites which received the required training.

### Outcome measures

#### Participant-level outcome measures

Participant-level outcome measures were measured at 12 weeks and 26 weeks post-injury, as outlined in the schedule in Table 1 (above). These include EuroQoL-5D-3L [17], mRS [18], FIM [19], HADS [21] and the Modified Caregiver Strain Index [20]. The mRS [18] score will be dichotomised into independent (0–2) or dependent / dead (3–6) and reported as a binary variable. Frequencies and percentages of the mRS [18] categories will be reported by trial phase (control/intervention). All other outcome measures will be treated as continuous; for these measures, the mean score will be reported together with the standard deviation.

#### System-level outcome measures

The median and IQR will be reported for occasions of service by trial phase (control/intervention).

##### Minimum processes of care (MPC)

The trial protocol lists twelve processes or care, not all of which are applicable to all participants (e.g. not all participants will require an interpreter). The achievement of minimum processes of care (MPC) is defined as the achievement of at least 80% of all process of indicators that are applicable to a participant. The achievement of MPCs will be reported as a binary variable (achieved/not achieved) at 12 and 26 weeks post-injury. Frequencies and percentages by trial phase (control/intervention) will be reported.

Details of how these outcomes will be presented are outlined in Table 4 (12-week outcomes) and Table 5 (26-week outcomes) of Appendix.

### Analysis of primary outcome

#### The primary effectiveness hypothesis states

Compared to UC, implementation of the proposed intervention package (IP) will result in a higher score on the Euro QOL–5D-3L [17] VAS at 26 weeks post-injury.

#### Analysis

A mixed effects linear regression model will be used to assess the between-group difference on Euro QOL–5D-3L [17] VAS score at 26 weeks post-injury. The model will control for injury type and severity (dichotomised mRS [18] at baseline), age at recruitment and time period (time since commencement of the trial) as fixed effects; recruitment site and participant/individual will be included as random effects. The treatment effect will be reported as the mean difference between the intervention condition and the control condition, together with the 95% confidence interval for the difference.

### Treatment of missing values

The primary analysis will be reported without imputation of missing data. If the amount of missing data warrants imputation (i.e. the number of missing values on the primary outcome measure exceeds 10%), missing data imputation will be conducted under the assumption that missing values are missing at random (MAR) [35]. That is, it is assumed that the values of the missing data may reasonably be predicted from all observed data. In particular, it will be assumed that missing values of the primary outcome measure (Euro QOL–5D-3L [17] VAS at 26 weeks post-injury) may be estimated from variables on which data has been collected (e.g. injury type, baseline injury severity, baseline mRS [18], age, gender, site, minimum processes of case, Euro QOL–5D-3L [17] VAS at 12 weeks post-injury), and on the observed values of Euro QOL–5D-3L [17] VAS at 26 weeks post-injury. To ensure robustness of the imputation, 20 imputed data sets will be generated with a separate model being developed for each imputation. These multiple imputations will be conducted using chained Eqs. [36, 37]. The pooled result of these imputed models will be reported and compared with the primary model (without imputed data).

### Sensitivity analyses

The imputation of missing values of the primary outcome is planned under an assumption of missing at random (MAR); therefore, a sensitivity of the results will be conducted to explore plausible departures from MAR.

The 2010 National Research Council Panel on the Handling of Missing Data in Clinical Trials [35] recommended a transparent and easily interpretable method for conducting a sensitivity analysis. Specifically, it recommended adding a parameter (delta) to the mean response. The parameter, delta, measures the degree of departure from missing at random. We propose using this approach to conduct a sensitivity analysis that assesses sensitivity of the results to plausible departures from the MAR assumption in this trial. If the inference about the treatment effects can be overturned by plausible values of the delta parameter, then the results of the trial will be considered equivocal.

### Secondary statistical hypotheses

The secondary effectiveness hypotheses (H2–H5) yield the following statistical hypotheses:

- H2.1 Compared to usual care, implementation of the IP will result in increased occasions of service in the first 12 weeks post-injury.
- H2.2 Compared to usual care, implementation of the IP will result in increased occasions of service across the first 26 weeks post-injury.
- H3.1 Compared to usual care, implementation of the IP will result in an increased proportion of participants receiving at least the minimum processes of care in the first 12 weeks post-injury.
- H3.2 Compared to usual care, implementation of the IP will result in an increased proportion of participants receiving at least the minimum processes of care across the first 26 weeks post-injury.
- H4.1 Compared to usual care, implementation of the IP will result in an increased proportion of participants achieving independence as determined by mRS [18] at 12 weeks post-injury.
- H4.2 Compared to usual care, implementation of the IP will result in an increased proportion of participants achieving independence as determined by mRS [18] at 26 weeks post-injury.
- H4.3 Compared to usual care, implementation of the IP will result in increased FIM™ [19] at 12 weeks post-injury.
- H4.4 Compared to usual care, implementation of the IP will result in increased FIM™ [19] at 26 weeks post-injury.
- H5.1 Compared to usual care, implementation of the IP will result in lower scores on the Modified Caregiver Strain Index [20] at 12 weeks post-injury.
- H5.2 Compared to usual care, implementation of the IP will result in lower scores on the Modified Caregiver Strain Index [20] at 26 weeks post-injury.
- H5.3 Compared to usual care, implementation of the IP will result in lower anxiety as measured on the Hospital Anxiety and Depression Scale [21] at 12 weeks post-injury.
- H5.4 Compared to usual care, implementation of the IP will result in lower anxiety as measured on the Hospital Anxiety and Depression Scale [21] at 26 weeks post-injury.
- H5.5 Compared to usual care, implementation of the IP will result in lower depression as measured on the Hospital Anxiety and Depression Scale [21] at 12 weeks post-injury.
- H5.6 Compared to usual care, implementation of the IP will result in lower depression as measured on the Hospital Anxiety and Depression Scale [21] at 26 weeks post-injury.

### Analysis of secondary statistical hypotheses

#### Occasions of service

A linear mixed effects regression model will be used to assess between-condition differences on occasions of service [H2]. Time period, age at recruitment, injury type and severity (dichotomised mRS [18] at baseline) will be controlled for as fixed effects in the model. Recruitment site and participant/individual will be included as random effects. In addition, time since injury and the interaction of time since injury with condition (control/intervention) will be included in the model. The interaction effect will be used to assess the between-group differences at 12 weeks post-injury [H2.1] and at 26 weeks post-injury [H2.2].

#### Processes of care

Minimum care processes [H3] will be dichotomised as achievement of minimum care processes (at least 80% of all applicable processes of care administered) versus non-achievement of minimum care processes. A mixed effects logistic regression model will be used to assess between-condition differences on this binary variable. Time period, age at recruitment, injury type and severity (dichotomised mRS^16^ at baseline) will be controlled for as fixed effects in the model. Recruitment site and participant/individual will be included as random effects. In addition, time since injury and the interaction of time since injury with condition (control/intervention) will be included in the model. The interaction effect will be used to assess the between-group differences at 12 weeks post-injury [H3.1] and at 26 weeks post-injury [H3.2].

#### Stroke disability/injury severity

The score on the mRS [18] will be dichotomised as independent (mRS [18] 0–2) or dependent /dead (mRS [18] 3–6). A mixed effects logistic regression model will be used to assess between-condition differences on this binary variable. Time period, age at recruitment, injury type and severity (dichotomised mRS [18] at baseline) will be controlled for as fixed effects in the model. Recruitment site and participant/individual will be included as random effects. In addition, time since injury and the interaction of time since injury with condition (control/intervention) will be included in the model. The interaction effect will be used to assess the between-group differences at 12 weeks post-injury [H4.1] and at 26 weeks post-injury [H4.2].

#### Functional independence

A linear mixed effects regression model will be used to assess between-condition differences on the Functional Independence Measure (FIM™) [19]. Time period, age at recruitment, injury type and severity (dichotomised mRS [18] at baseline) will be controlled for as fixed effects in the model. Recruitment site and participant/individual will be included as random effects. In addition, time since injury and the interaction of time since injury with condition (control/intervention) will be included in the model. The interaction effect will be used to assess the between-group differences at 12 weeks post-injury [H4.3] and at 26 weeks post-injury [H4.4].

#### Caregiver strain

Data on the Modified Caregiver Strain Index [20] was obtained from 25 next-of-kin at 12 weeks post-injury and 21 next-of-kin at 26 weeks post-injury. Given the relatively small sample size for these data, differences in caregiver strain between the control and intervention phases of the trial will be assessed using independent samples *t*-tests. The *t*-tests will be conducted at the 5% significance level, and 95% confidence intervals will be reported. No additional modelling will be undertaken and no adjustments for any covariates will be made.

#### Anxiety and depression

Separate linear mixed effects regression model will be used to assess between-condition differences on anxiety and depressions scales of the HADS [21]. Time period, age at recruitment, injury type and severity (dichotomised mRS [18] at baseline) will be controlled for as fixed effects in the models. Recruitment site and participant/individual will be included as random effects. In addition, time since injury and the interaction of time since injury with condition (control/intervention) will be included in the models. The interaction effect will be used to assess the between-group differences at 12 weeks post-injury [H5.3] and at 26 weeks post-injury [H5.4].

### Safety assessments

Adverse events (AEs) are classified as possibly, probably or definitely attributable to the intervention. Serious adverse events (SAEs) are classified as not related, unlikely, possibly, probably or definitely attributable to the intervention as they occur (between consent and 26 weeks post brain injury). AEs and SAEs will be reported by condition (control/intervention) in the main paper. The structure of this table is presented in Table 6 of Appendix.

AEs and SAEs possibly, probably or definitely attributable to the intervention are expected to be rare. Therefore, no formal hypotheses are associated with these. As rare events, AEs and SAEs are expected to have a Poisson or a negative binomial distribution. If the data suggests that there is a between-condition difference of more than 3 standard deviations in AEs or SAEs, the distribution will be modelled, and an appropriate generalised linear model will be developed to assess between-condition differences.

## Changes since the commencement of the trial

### Recruitment rate

Recruitment remained below the anticipated rate throughout the trial. This was due to a number of issues including:

- The COVID-19 pandemic from 2020 to the trial close;
- Difficulty experienced in recruitment of people with TBI;
- A hiatus in the recruitment of participants incapable of independent consent, i.e. discontinuation of approval to gain consent by proxy from 2018 to 2020 as part of the Western Australian Guardianship and Administration Amendment (Medical Research) Act 2020 [38];
- Initial ethical approval for recruitment within hospital setting only—participants were often discharged before being recruited; even after approval was gained to recruit within the community, this remained problematic as many were rural residents, and recruiting remotely proved to be challenging; and
- Limited availability of recruiters in some hospitals at times, e.g. staffing shortages.

All assessments in this trial were designed to be undertaken remotely or in person and are validated for remote administration. Similarly, the Aboriginal Brain Injury Coordinator services were able to be undertaken via telephone and telehealth, as planned. Therefore, COVID-19 pandemic, which was declared as a global pandemic on March 23, 2020, did not affect the administration of the trial for recruited participants. Recruitment concluded on 31 July 2021, as planned. The total number of patients recruited was 108 with uneven distribution across sites and clusters. The numbers recruited by site/cluster per time period are presented in Table 7 in Appendix.

### Effect of lower sample size on statistical analysis plan

The final sample size of 108 was approximately one-third the estimated minimum required sample size of 312. As a result, one or more of our proposed mixed effects regression models (linear or logistic) may fail to converge. Nevertheless, we will attempt to fit the models as proposed in the first instance. If the model for an outcome measure fails to converge, we will approach the modelling of that outcome by removing effects from the model in the following order:

1. Time period
2. mRS [18]
3. Injury severity
4. Injury type
5. Site

If the model for an outcome still fails to converge, the outcome may be modelled with separate models at each timepoint: 12 weeks or 26 weeks. In this case, time since injury and interaction between time since injury and condition (control/intervention) will be redundant. The other effects will be included in the model using a forward selection strategy to achieve the best model based on the Akaike Information Criterion (AIC). The assumptions for all statistical models will be assessed graphically using residual plots.

### Current trial status

Recruitment to Healing Right Way finished July 31, 2021, with final follow-up of participants completed Jan 31, 2022. Data lock is anticipated to be March 18. The final version of the statistical analysis plan was approved by all members of the research team on March 8, 2022.

## Figures and Tables

**Table 7:**
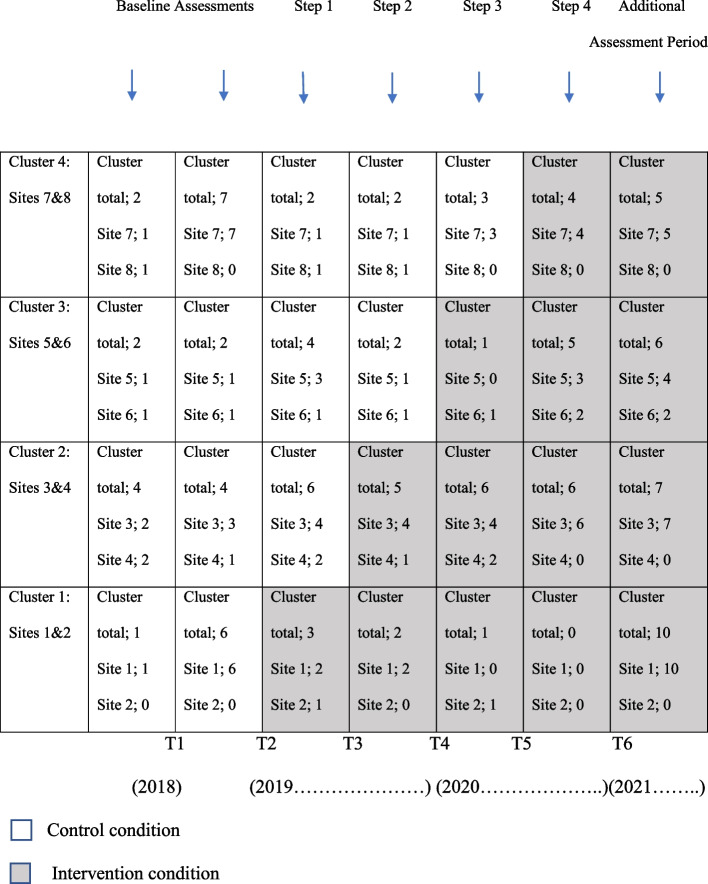
Actual recruitment numbers

## Data Availability

Group data generated and analysed related to the primary outcome measures will be publicly available in the main study results publication. Due to the data involving Aboriginal persons, we currently do not have ethics approval to make public individual-level data even when de-identified.

## Appendix

Tables 3, 4, 5, 6 and 7

**Table 3:** Participant demographics and baseline clinical characteristics

	**Control**	**Intervention**	**All**
	*n* (%)	*n* (%)	*n* (%)
**Recruitment region**			
Metropolitan			
Regional			
**Patient details**			
Age median (IQR)			
< 65			
65–80			
> 80			
Gender			
Male			
Female			
Other/not disclosed			
**Place of residence prior to brain injury**			
Urban area			
Inner regional			
Outer regional			
Remote			
Very remote			
**Living arrangements prior to brain injury**			
Home (alone)			
Home (with others)			
Supported accommodation, e.g. nursing home, hostel			
No fixed home			
Other			
**Injury type**			
TBI			
Stroke			
**Injury severity**			
Mild			
Medium			
Severe			
**Baseline mRS** (binary)			
Independent (0–2)			
Dependent/dead (3–6)			
**Baseline HADS**			
Anxiety			
Depression			
**Baseline FIM**			
**Alcohol consumption**			
**Drug use (Yes)**			
**Diabetes (Yes)**			
**Heart disease (Yes)**

**Table 4:** Outcomes at 12 weeks

	**Control**	**Intervention**	**All**
**Primary outcome measure**			
EuroQoL-5D-3L			
**Secondary participant-level outcomes**			
mRS			
FIM™			
HADS: Anxiety			
HADS: Depression			
Participant satisfaction			
Modified Caregiver Strain Index			
**System-level outcomes**			
Minimum Processes of Care Achieved (Yes)			
Occasions of Service

**Table 5:** Outcomes at 26 weeks

	**Control**	**Intervention**	**All**
**Primary outcome measure**			
EuroQoL-5D-3L			
**Secondary participant-level outcomes**			
mRS			
FIM™			
HADS: Anxiety			
HADS: Depression			
Participant satisfaction			
Modified Caregiver Strain Index			
**System-level outcomes**			
Minimum Processes of Care Achieved (Yes)			
Occasions of Service

**Table 6:** Adverse events and serious adverse events

	**Control**	**Intervention**	**All**
	*n* (%)	*n* (%)	*n* (%)
No. of adverse events (AEs)			
Neurological complications			
0			
1			
2			
> 2			
No. of serious adverse events (SAEs)			
0			
1			
2			
> 2			
